# 15 years of PhosphoSitePlus^®^: integrating post-translationally modified sites, disease variants and isoforms

**DOI:** 10.1093/nar/gky1159

**Published:** 2018-11-16

**Authors:** Peter V Hornbeck, Jon M Kornhauser, Vaughan Latham, Beth Murray, Vidhisha Nandhikonda, Alex Nord, Elżbieta Skrzypek, Travis Wheeler, Bin Zhang, Florian Gnad

**Affiliations:** 1 Department of Bioinformatics and Computational Biology, Cell Signaling Technology Inc., Danvers, MA, USA; 2 University of Montana, Missoula, MT, USA

## Abstract

For 15 years the mission of PhosphoSitePlus^®^ (PSP, https://www.phosphosite.org) has been to provide comprehensive information and tools for the study of mammalian post-translational modifications (PTMs). The number of unique PTMs in PSP is now more than 450 000 from over 22 000 articles and thousands of MS datasets. The most important areas of growth in PSP are in disease and isoform informatics. Germline mutations associated with inherited diseases and somatic cancer mutations have been added to the database and can now be viewed along with PTMs and associated quantitative information on novel ‘lollipop' plots. These plots enable researchers to interactively visualize the overlap between disease variants and PTMs, and to identify mutations that may alter phenotypes by rewiring signaling networks. We are expanding the sequence space to include over 30 000 human and mouse isoforms to enable researchers to explore the important but understudied biology of isoforms. This represents a necessary expansion of sequence space to accommodate the growing precision and depth of coverage enabled by ongoing advances in mass spectrometry. Isoforms are aligned using a new algorithm. Exploring the worlds of PTMs and disease mutations in the entire isoform space will hopefully lead to new biomarkers, therapeutic targets, and insights into isoform biology.

## INTRODUCTION

Protein post-translational modifications (PTMs) are enzyme-catalyzed covalent additions to side-chains of amino acids of proteins (1). The highly dynamic process of post-translational modification within a cell forms a complex and ever-changing nexus of protein modifications that respond to autonomous and exogenous signals. Dynamic networks of PTMs regulate the fundamental processes of life including transcription, metabolism and cell division. The central role that PTMs play in cellular signaling has made it essential to have resources that provide researchers with accurate and comprehensive information about PTMs. Such resources need to include not only an accurate syllabary of the modification sites and surrounding sequences across species, but also the upstream and downstream biological processes and molecular functions that are impacted by the modification state of the post-translationally modifiable site.

PhosphoSitePlus^®^ (PSP, https://www.phosphosite.org) is such a resource. It is focused on the three most commonly studied organisms in biomedical research: human, mouse and rat. Information about experimentally observed PTMs, manually curated by expert scientists, includes upstream regulation by treatments, ligands, and enzymes, and downstream regulation of molecular, cellular and biological consequences controlled by the PTM (2).

While there are over 400 known PTMs (3), the major classes of modifications that are curated into PSP include those that are most widely studied in the regulation of intracellular signaling: phosphorylation of serine, threonine and tyrosine; ubiquitylation and ubiquitin-like modifications of lysine; acylation of lysine (e.g. acetylation and succinylation); and three distinct types of methylation on lysine (mono-, di- or trimethyl-lysine) and on arginine (dimethyl [asymmetric or symmetric], or monomethyl-arginine). These PTMs are dynamically regulated in an emergently complex and coordinated fashion by cellular enzymes that add or remove them from proteins. Regulatory PTMs control cellular processes, molecular functions, or changes in subcellular localization. Each of these modification types can co-occur within close proximity, or overlapping with, each other on the same protein, significantly modifying the biological meaning of each PTM alone (4).

The significant impact of PTM events on cell functions and processes is very evident from their involvement in diseases. Perturbed signaling is the most common cause of uncontrolled cancer-triggering cell growth and proliferation, and other disease-associated cellular malfunctions. Recent advances in next-generation sequencing technologies set the stage for gigantic initiatives such as The Cancer Genome Atlas (TCGA) (5) to profile large numbers of tumors, or the 1000 Genomes project (6) to catalog human genetic variations. Analyses of these data have shown that transferases, including kinases (7) and epigenetic regulators (8), are amongst the most commonly mutated proteins in disease. However, to fully comprehend the interplay between DNA mutations and pathological phenotypes, the integration of genomic with proteomic data is required (9).

The goal of many of the new features of PhosphoSitePlus is to provide information and visualization tools that may enable researchers to identify potentially functional sites and help distinguish sites that regulate pathogenesis from sites of lesser biological consequence. Multiple types of evidence in PSP can help identify functionally important sites including: the evolutionary conservation of a modification site (10,11); the frequency with which a PTM has been observed in unbiased tandem mass spectrometry (MS2) experiments, which correlates with the conservation of the site (12); and the intersection between PTMs and disease associated missense mutations, which may provide a framework for interpreting key signaling networks and associated PTMs that are perturbed by pathogenic mutations.

## CONTENT GROWTH AND CHANGES SINCE 2015

### Statistics: sites, organisms, articles, journals, curation and disease mutations

#### Site statistics

The total number of modification site groups (homologous sites across all species are counted just once) curated into PSP as of September 2018 is 451 453, up 30% from 2015 (Table 1). While phosphoryl, ubiquityl and acetyl represent over 90% of the modification types in PSP (65%, 17% and 8%, respectively), the largest increases in the proportion of sites curated over last three years have been sumoyl, monomethyl and ubiquityl (10-fold, 3-fold and 1.5-fold increases, respectively).

**Table 1.** Total number of post-translationally modified site groups in PSP: 2015 versus 2018

Modification type	2015	2018	fold-increase
Phosphorylation			
Serine	144 899	175 101	1.21
Threonine	61 654	72 486	1.18
Tyrosine	41 273	46 310	1.12
Ubiquityl-Lys	51 258	77 372	1.51
Acetyl-Lys	27 660	38 387	1.39
Methylation			
Monomethyl-Arg	5000	15 191	3.04
Dimethyl-Arg	2556	2860	1.12
Trimethyl-Lys	321	343	1.07
Sumoyl-Lys	816	8546	10.47
Succinyl-Lys	0	4627	
*N*-glyco-Asn	0	6398	
*O*-galNAc-Ser/Thr	2118	2118	1.00
*O*-glcNAc-Ser/Thr	1393	1726	1.24
**Total sites:**	**338 948**	**451 453**	**1.33**

#### Organisms

PSP is focused on the three most commonly studied species in biomedical research, human, mouse and rat. These organisms account for 63.9%, 27.2% and 8.6% of the sites curated into PSP. While 23 other species are represented in PSP including 15 mammals, three birds, and one each of Asteroidea, ray, insect and amphibian, they together constitute just 0.3% of the total sites aggregated in PSP.

#### Protein groups: proteins and isoforms

The sequence space of PSP is composed of 56 820 protein sequences contained within 22 944 protein groups. Of these groups, 2532 have no associated PTMs in PSP. When the new Mirage sequence alignment algorithm (https://github.com/TravisWheelerLab/Mirage) is in place (discussed below in the ‘Future Directions’ section), sequences will be synchronized with UniProtKB/Swiss-Prot (13) quarterly. Because of the provisional nature of TrEMBL sequences, including some that cannot be explained by in-frame or out-of-frame sequences, most will be excluded from PSP. Previously, individual sequences were updated as needed, on a daily or weekly basis, a process that is untenable for efficient high throughput curation.

As of September 2018, there are only 6445 human and mouse isoforms in PSP from 4206 protein groups, a number that is adequate to account for all MS2 peptides heretofore curated into PSP. We are in the process of importing all human and mouse UniProtKB/Swiss-Prot isoforms into PSP - this includes 22 418 human and 8743 mouse isoforms from 10,644 protein groups. The human and mouse isoforms will be aligned using the Mirage multiple sequence alignment (MSA) software package as described in the ‘Future Directions’ below.

#### Articles and journals curated into PSP

The total number of articles curated into PSP is 22 333 (September 2018), including 22 075 articles describing low-throughput characterization (LTP) of PTMs, and 258 that used high throughput (HTP) mass spectrometry to identify PTMs. Over the last three years, 4201 articles, 94 of which report HTP data, were curated into PSP. These articles are from 439 separate journals and, as in all previous periods, more papers were curated from the journal J. Biol. Chem than any other (455 articles, or 11%).

Open access journals (OAJ) are trending as the fastest growing sector of articles being curated into PSP. Six years ago, no OAJ was in the top 5, while in the 2015–2018 period, two OAJs are in this group: Oncotarget and PloS One. They have risen to positions 2 and 3, respectively, with 382 and 285 papers each, for a total of 16%. Positions 4 and 5 are held by *Oncogene* and *Proc. Natl. Acad. Sci. U.S.A*., with 241 and 181, for a total of 10% of the papers curated in the last 3 years.

#### External links

A link has been added in the ‘Cross-references’ section of the Protein Information page to the cBioPortal for Cancer Genomics (cbioportal.org) (14,15), a resource enabling users to visualize and analyze large-scale cancer genomics datasets from sources including the TCGA Research Network (cancergenome.nih.gov).

#### Quantitative data

Curation of HTP records during the last three years has focused almost exclusively on datasets that include quantitative values for peptides/sites. A total of 79 quantitative HTP records have been curated into PSP to date.

#### Curation of data

Information from articles using LTP methodologies is manually curated via the curatorial interface. Metadata curated from LTP papers includes upstream and downstream regulatory information. The information in HTP site tables has been formatted manually prior to scripted submission of the peptides, sites and quantitative data to the database. Metadata from HTP submissions are manually curated.

#### Disease informatics

Proteins associated with inherited diseases have been retrieved from ClinVar (16), which lists 3265 proteins with missense mutations that are ‘pathogenic’ or ‘pathogenic/likely pathogenic’ in their July 2018 release.

#### Crosstalk between inherited disease mutations and post-translational modifications

Mutations that change the amino acid of a residue that is post-translationally modified can disrupt, or alter, the cellular signals that are relayed by that PTM. Such mutations, when they occur on or within 5 residues of the modified site, are called PTMVars (12), and include Class I variants which change the amino acid at the site of a PTM, and Class II variants, which occur within ±5 residues of the PTM. Both types can profoundly alter the signals that that are relayed via that PTM. We have combined PTM site data from PSP with the germline missense mutations to give users another dimension in which to evaluate post-translational modifications.

Human germline missense mutations included in PSP as of autumn 2018 are from the UniProtKB Humsavar dataset (www.uniprot.org/docs/humsavar) and are updated monthly. The June 2018 release includes 30 191 disease variants: over 18 500 have been assigned dbSNP IDs. These are expressed on 2532 human proteins and are associated with >3000 inherited diseases and syndromes.

Systematic analysis showed that 420 genetic variants overlap with 402 PTM sites on 276 proteins. Table 2 lists 10 such PTM sites that have been mutated, but about which there appear to be no LTP publications that describe the biological activity of the modified site, but with which many HTP records are associated. Supplemental Table S1 lists 70 Class I mutations including 63 phosphorylation, 6 acetylation and 1 ubiquitylation sites. Table 1 summarizes the ten Class I PTMVars that have the greatest number of associated HTP but no LTP records. At the top of the list is phosphoTyr62 SHP-2: Tyr62, when mutated, is associated with Juvenile Myelomonocytic Leukemia (17) and Noonan Syndrome 1 (18). Five of these variants overlap with PTM sites. One, phospho-Y62, has been detected in 2,090 mass spectrometry analyses (Table 2) including 800 patient tumors: 36% are lung, 13% liver and 8% ovarian cancers.

**Table 2.** Missense mutations that replace frequently modified residues

GENE	VAR	TYPE	LTP	HTP	DISEASE
PTPN11	Y62D	ph	0	2116	Noonan syndrome 1, Familial
LDLR	Y82C	ph	0	164	Familial hypercholesterolemia
PKP2	Y63C	ph	0	112	Ventricular dysplasia, arrhythmogenic
BTK	Y36C	ph	0	111	X-linked agammaglobulinemia
TUBB	Y22F	ph	0	104	Skin creases, circumferential
ACTC1	Y16C	ph	0	92	Cardiomyopathy, hypertrophic
KRT10	Y16D	ph	0	79	Epidermolytic hyperkeratosis
GNAS	K33N	ub	0	47	Pseudohypoparathyroidism
UPF3B	Y16D	ph	0	38	Mental retardation
HMGCL	K48N	ac	0	45	3-hydroxy-3-methylglutaryl-CoA lyase deficiency

GENE, HGNC gene names; VAR, germline disease missense mutations from Humsavar 20 June 2018, (www.uniprot.org/docs/humsavar); LTP, number of low-throughput references curated into PSP; HTP, number of high-throughput references curated into PSP; TYPE, modification type: PH (phosphorylation), AC (acetylation), UB (ubiquitylation); and DISEASE, associated disease from Humsavar.

In addition to SNPs that change the amino acids at PTM sites, 3778 genetic variants occur in flanking regions (+/- five residues) of 4147 PTM sites on 1113 proteins. For example, phosphoglycerate mutase 2 (PGAM2) contains three SNPs (E89A, R90W, G97D) in the flanking sequence of Y92, all of which have been associated with glycogen storage disease 10 (19). Phospho-Y92 has been reported in 2556 records, but no biological function has been attributed to it to our knowledge. Y92 and its surrounding sequence are parts of a substrate binding sequence in yeast (20), suggesting that the phosphorylation of Y92 in humans may reflect a mechanism that regulates substrate binding in PGAM2 throughout a substantial period of evolutionary time.

#### Somatic cancer mutations

Somatic cancer mutations used in PSP are derived from the 15 published TCGA datasets (5) which are listed in the ‘Cancer’ tab on the Protein Page (Fig 2C) and are available from cBioPortal (14,15). These datasets include 481 370 mutations from 4,440 tumors across 15 cancer types. The cancer types included are Leukemia (AML), Bladder (BLCA), Breast (BRCA), Colorectal (COADREAD), Glioblastoma (GBM), Head & Neck (HNSC), Kidney (KICH and KIRC), Lung (LUAD and LUSC), Ovarian (OV), Prostate (PRAD), Stomach (STAD), Thyroid (THCA) and Endometrial (UCEC).

Most of these are passenger mutations. We therefore selected only hotspot mutations detected in three or more tumors, since recurrent mutations are likely cancer-driving and present the most characteristic feature of oncoproteins (8,21). Notably, the number of tumors per TCGA cancer type varied from 65 (chromophobe renal cell carcinoma) to 817 (breast cancer). Thus, the selection of hotspot mutations may be biased towards cancer types with larger cohorts in the associated TCGA study. Overall, 62 of the 2700 hotspot mutations intersect with 64 PTM sites in 48 proteins. For example, 34 studies found evidence for phosphorylation of Raf1 on residue S257. A total of five tumors types showed a mutation of this residue. Similarly, we found interesting cases for potential oncogenic rewiring of signaling networks due to changes in kinase sequence motifs. Overall, 732 hotspot mutations reside in flanking regions of PTM sites. For example, three tumors show a mutation of growth factor receptor-bound protein 10 (GRB10) on residue T422. This mutation directly intersects with phospho-T422 and is also proximate to phospho-S418 and phospho-S426. All of these phosphosites have been detected in multiple studies, but no associated biological activity has been reported to our knowledge.

The germline and somatic nsSNPs included in PSP will change over time as additional disease associated mutations are discovered, as we understand with more clarity which mutations are closely linked to pathogenesis, and as we incorporate data from additional resources. For example, we may incorporate a subset of missense mutations from 18 additional cancer types in the TCGA PanCancer Atlas (22).

#### Performance, cloud migration and dynamic visualization

To improve the user experience, we optimized a number of queries and pre-calculated associated statistics for some expensive queries including the HTP and LTP counts. Query optimization speeded up single protein searches by a factor of 15. Now it takes an average of less than two seconds to retrieve information for a specified protein. The web application and database were moved to the cloud in 2018, making future upgrades and extensions more straightforward. The dynamic visualization of PTM sites and nsSNPs in the Lollipop Plot on the Protein Page was programmed using JavaScript and D3.

## NEW FEATURES OF THE USER INTERFACE

Major interface makeovers of the ‘Homepage’ and the ‘Protein Page’, and a new disease search and functionality have been added in 2018.

### Homepage

**Figure 1.**
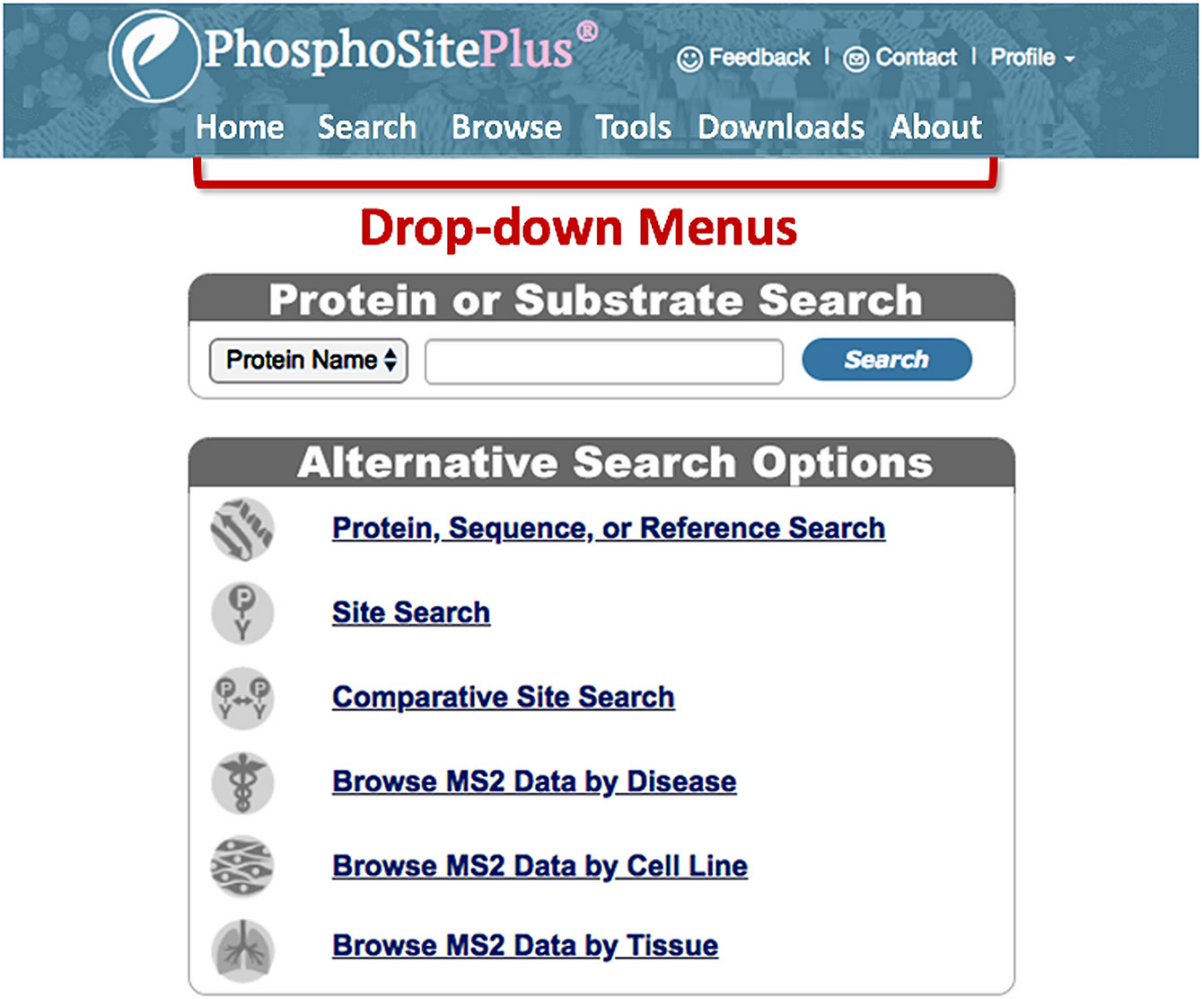
Visual simplicity of the new Homepage. Most of the text and features on the old Homepage have been moved or removed from the new Homepage. Two sets of searches, the ‘Protein or Substrate Search’, and the six searches in the ‘Advanced Search and Browse Section’ are still accessed directly from the Homepage. The ‘Site Statistics’ table has been moved to the ‘About’ menu, and the Motif and Sequence Logo tools have been moved to the ‘Tools’ menu.

The Homepage has been transformed from a busy page loaded with content to a sleek and parsimonious design (Figure 1). Functionalities previously on the Homepage have been moved to the ‘Drop-down Menus’ at the top of the page: ‘Motif’ and ‘Sequence Logo’ tools to the ‘Tools’ menu; and multiple informational features, including ‘Site Statistics’ have been moved to the ‘About’ menu. Two sets of basic queries remain on the Homepage: the ‘Protein or Substrate Search’ window and the ‘Alternative Search Options’. These remain the same as previous iterations of PSP, with the exception of the disease search, discussed in greater detail below.

### Search for disease associated proteins

**Figure 2.**
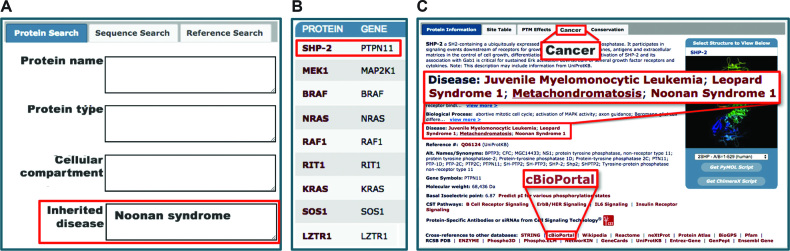
Find proteins in which missense mutations are associated with specific diseases or syndromes. (**A**) Disease query on the ‘Protein Sequence and Reference Search’ Page. (**B**) ‘Search Results’ lists proteins in which mutations are causally associated with the selected pathological state.Clicking on the protein selected in the ‘Search Results’ goes to (C) the ‘Protein Page’, where the ‘Protein Information’ section lists additional diseases or syndromes with associated links. The ‘Cancer’ tab opens a PSP page displaying statistics about the number of somatic mutations from TCGA observed for this protein.

A new search for proteins that are known to be causally associated with inherited diseases and syndromes has been added to the Protein Sequence and Reference Search interface (Figure 2). A search for proteins associated with ‘Noonan Syndrome’ (Figure **2**A) returns nine proteins associated with the syndrome (Figure 2B). Clicking on SHP-2 opens its Protein Page. The ‘Protein Information’ window is one of the five tabbed windows on the ‘Protein Page’ (Figure 2C). The names of three inherited diseases in addition to Noonan syndrome associated with SHP-2 are displayed: Juvenile Myelomonocytic Leukemia, Leopard Syndrome 1, and Metachondromatosis. Clicking on one of the listed diseases will open up the corresponding disease page in either OMIM^®^ (omim.org/entry/163950) (23) or MedGen (www.ncbi.nlm.nih.gov/medgen/22527) (24). Furthermore, the ‘cancer tab’ summarizes mutation frequencies of the given protein across different cancer types. Links to the corresponding TCGA publications are provided at the bottom of the page.

### Redesigned site tables

**Figure 3.**
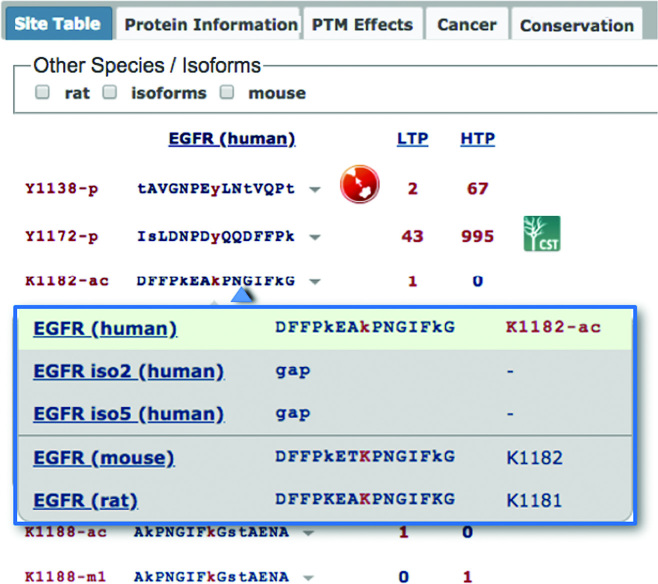
Redesigned Site Tables. Site Tables are found in one of the five tabs on the Protein Page. The sites and sequences from the selected species are displayed sequentially down the page. Clicking on a sequence opens a dropdown window displaying the sequences of homologous sites across all members of the protein group. Additional columns for other species or isoforms can be added by selecting the checkboxes above the site table. Effects of the PTM site on biological processes or the protein itself are shown by clicking on the icon displayed after the site sequence. Blue icons to the right of the number of HTP records lists site-specific antibodies available from Cell Signaling Technology.

Site Tables have been moved to one of the five tabs on the Protein Page (Figure 3). Previously, sites from all species were shown in the Site Table. In the new version, only the sites from the selected species show up by default. PTM sites, their flanking sequences and LTP & HTP counts, are listed sequentially down the page. Residue numbers and flanking sequences are specific to the selected species, while the numbers in the LTP & HTP columns include the number of curated records for all orthologous members of a site group. Clicking on a sequence opens a dropdown window displaying the sequences of the homologous sites across all members of the protein group (Figure 3), allowing users to investigate conservation of the site and flanking sequence within a column instead of a row. Sequences from other species or isoforms can be displayed as additional columns via the checkboxes on top of the Site Table.

Additionally, effects of the PTM site on protein function or biological processes can be shown by clicking on the icon displayed after the site sequence. These are also summarized in a separate ‘PTM Effects’ tab (Figure 3). As in previous iterations of PSP, blue icons to the right of the number of HTP records indicate antibodies specific to the site that are available from Cell Signaling Technology.

### Interactive visualization of PTM sites

**Figure 4.**
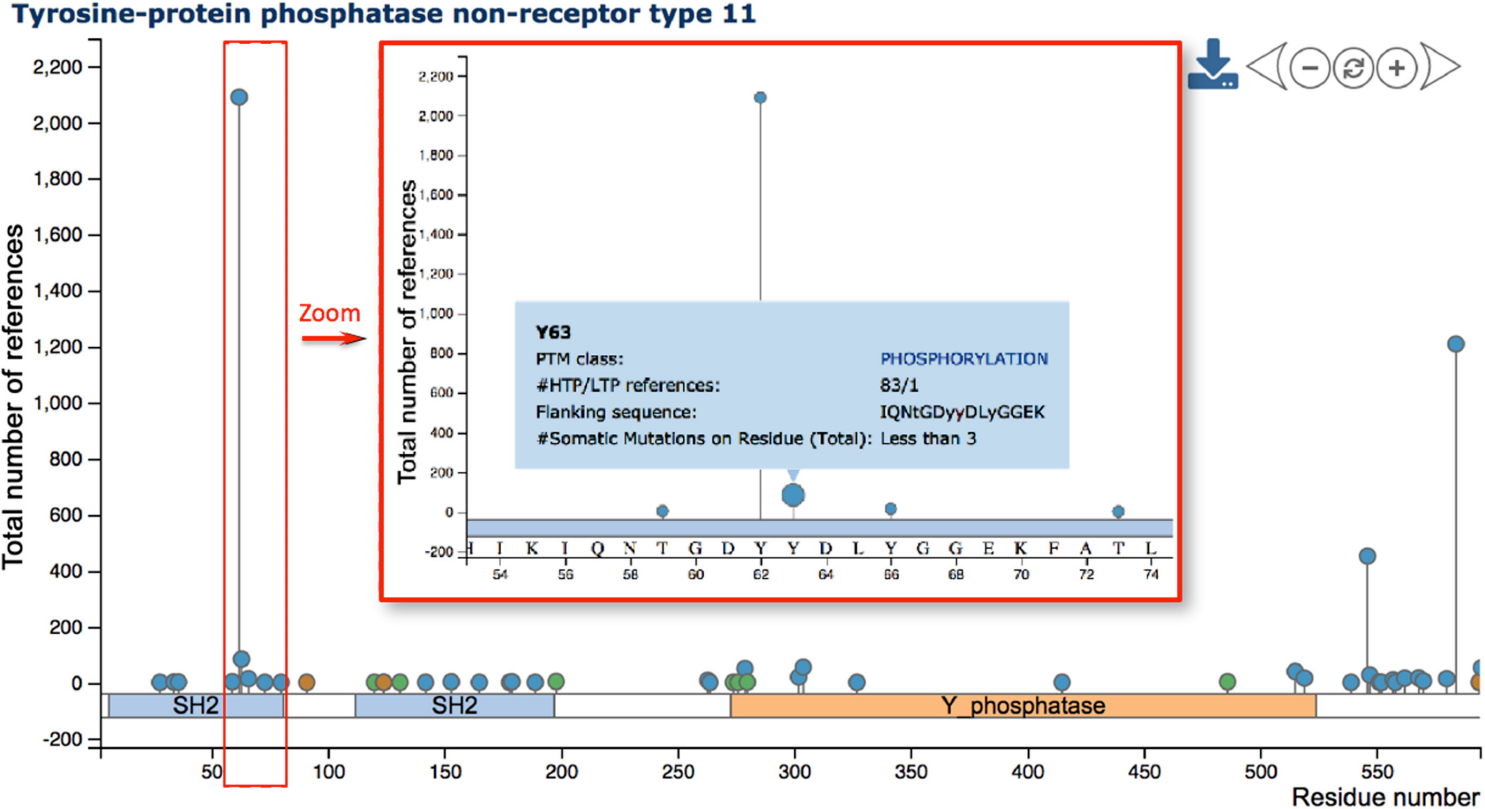
Visualizing PTM sites in lollipop plots. The lollipop plot illustrates phosphorylation (blue), acetylation (green), ubiquitylation (brown) and other (gray) PTM sites on human SHP-2. The horizontal axis displays protein residues and domains, while the vertical axis reflects the number of HTP and LTP papers reporting specified PTM sites of SHP-2. Zooming in (via buttons or mouse) allows the illustration of corresponding amino acids. The plot also shows details about PTM sites (here: phospho-Y63) triggered by hovering the mouse over lollipops.

PTM sites are visualized in a dynamic and novel way: ‘lollipop plots’ allow users to interactively explore identified PTM sites along with quantitative data at the residue level (Figure 4). The horizontal axis of a lollipop plot describes residues and domain annotations from Pfam (25) for a given protein. A zoom function allows users to focus on specific regions of interest. Clicking the ‘+’ button, using the mouse wheel/multi touch surface, or double-clicking on the plot can trigger the zooming in function. At a certain zoom level, the horizontal axis displays residue numbers and amino acids (Figure 4). Mouse dragging (selecting the plot and moving the mouse while keeping the mouse button pressed) or clicking the ‘left’ or ‘right’ buttons allows users to explore along the protein sequence. Analogous to zooming in, users may zoom out or reset the zoom level by clicking the ‘reset’ button. In addition to protein sequence and domain annotation, ‘lollipops’ represent identified PTM sites and reside along the axis. Colors reflect the different PTM classes. Hovering the mouse over these lollipops triggers the display of a tooltip (‘hover box’) with additional information about the PTM site including associated literature and flanking sequence.

While the horizontal axis presents protein features including PTM sites along the sequence, the vertical axis shows associated quantitative data. By default, the vertical axis reflects the number of LTP and HTP studies that reported the identification of a given PTM site. The control panel on the right enables users to distinguish between LTP and HTP evidence, or to apply log2 transformation. Moreover, one may select PTM sites reported in five or more studies. In summary the introduction of lollipop plots enables the user to interactively explore the landscape of PTM sites. The current view of the plot can be saved as high-resolution image via the download button. The minimize button makes the lollipop plot disappear.

### Adding disease mutations to lollipop plots

**Figure 5.**
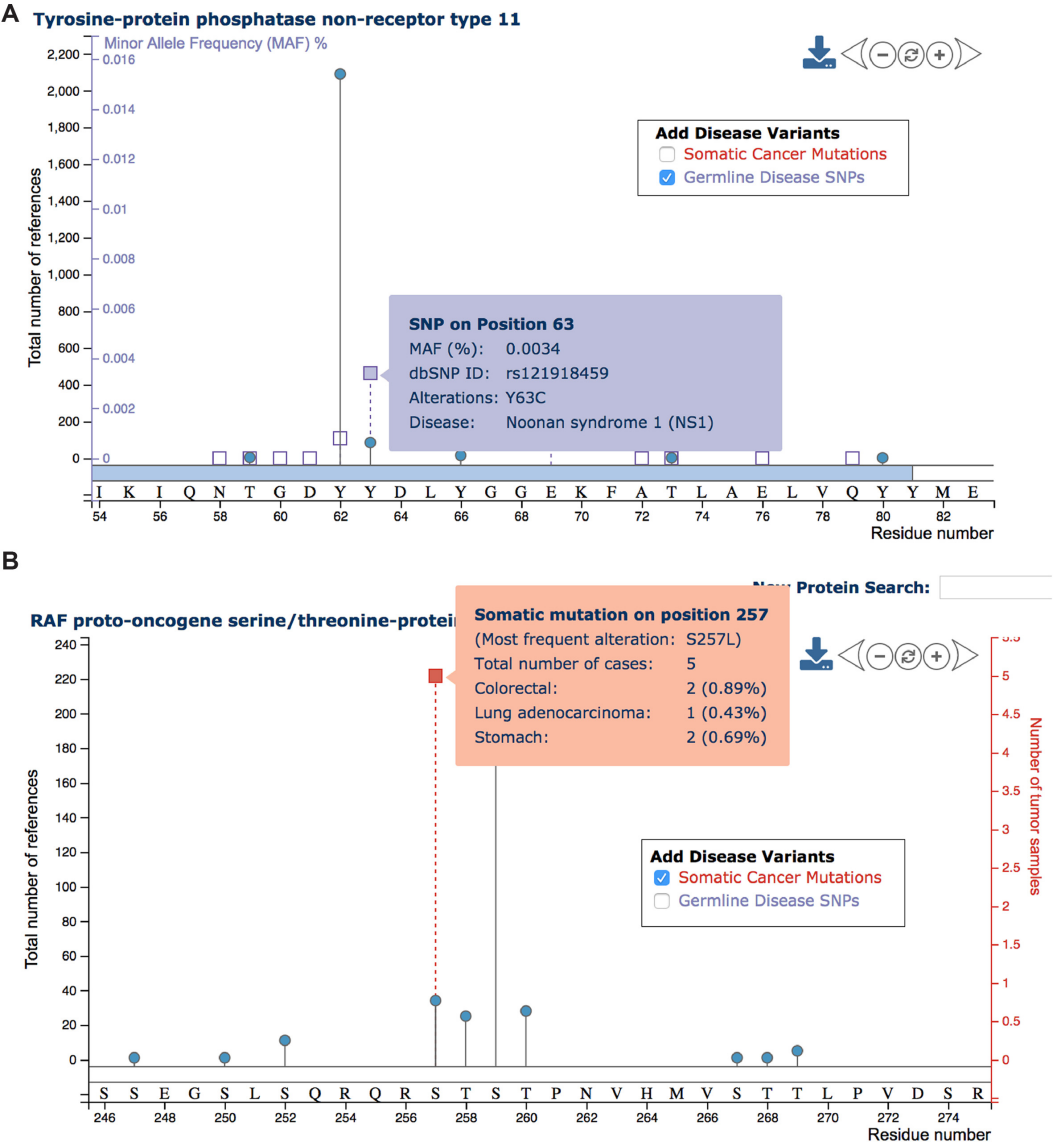
Adding disease mutations to lollipop plots. (**A**) The lollipop plot displays PTM sites and disease-associated SNPs (purple squares) on human SHP-2. The inclusion of disease-associated SNPs yields an additional purple vertical axis reflecting the minor allele frequency (MAF), if available. Hovering the mouse over squares yields the display of further details about the disease SNP. (**B**) Analogously, hotspot mutations from TCGA can be visualized as red squares. In this case the additional vertical axis (red) reflects the total number of tumors having the specified mutation.

To visualize the proximity of PTM sites and disease variants, lollipop plots provide the option to add mutations to the visualization. Irrespective of their proximity to PTM sites, disease-associated SNPs can be included as purple squares (Figure 5A). An additional vertical axis reflects corresponding minor allele frequencies (MAFs) for these SNPs, if available. Hovering the mouse over SNPs provides detailed information about associated diseases. Analogously, somatic hotspot mutations from TCGA can be visualized in lollipop plots. These appear as red squares, and hovering the mouse over gives information about the mutation frequency in the combined panel of 15 published TCGA cancer types (Figure 5B). Adding TCGA mutations to the lollipop plot also yields an additional vertical axis, which reflects the number of associated tumors.

## FUTURE DIRECTIONS AND DISCUSSION

### Short term

#### Splice-aware multiple sequence alignment

Alternative isoform usage plays important roles in many biological processes (26,27). Splicing-factor alterations and aberrant isoform expression are implicated in a growing number of diseases including cancer (28). In order to support research into isoform regulation and expression, we are in the process of importing over 30 000 UniProtKB/Swiss-Prot human and mouse isoform sequences into PSP, and all must be accurately aligned with other members of the family. Standard multiple sequence alignment (MSA) tools are adequate for aligning paralogs and homologs, but not for the accurate alignment of multiple isoforms within a protein family.

**Figure 6.**
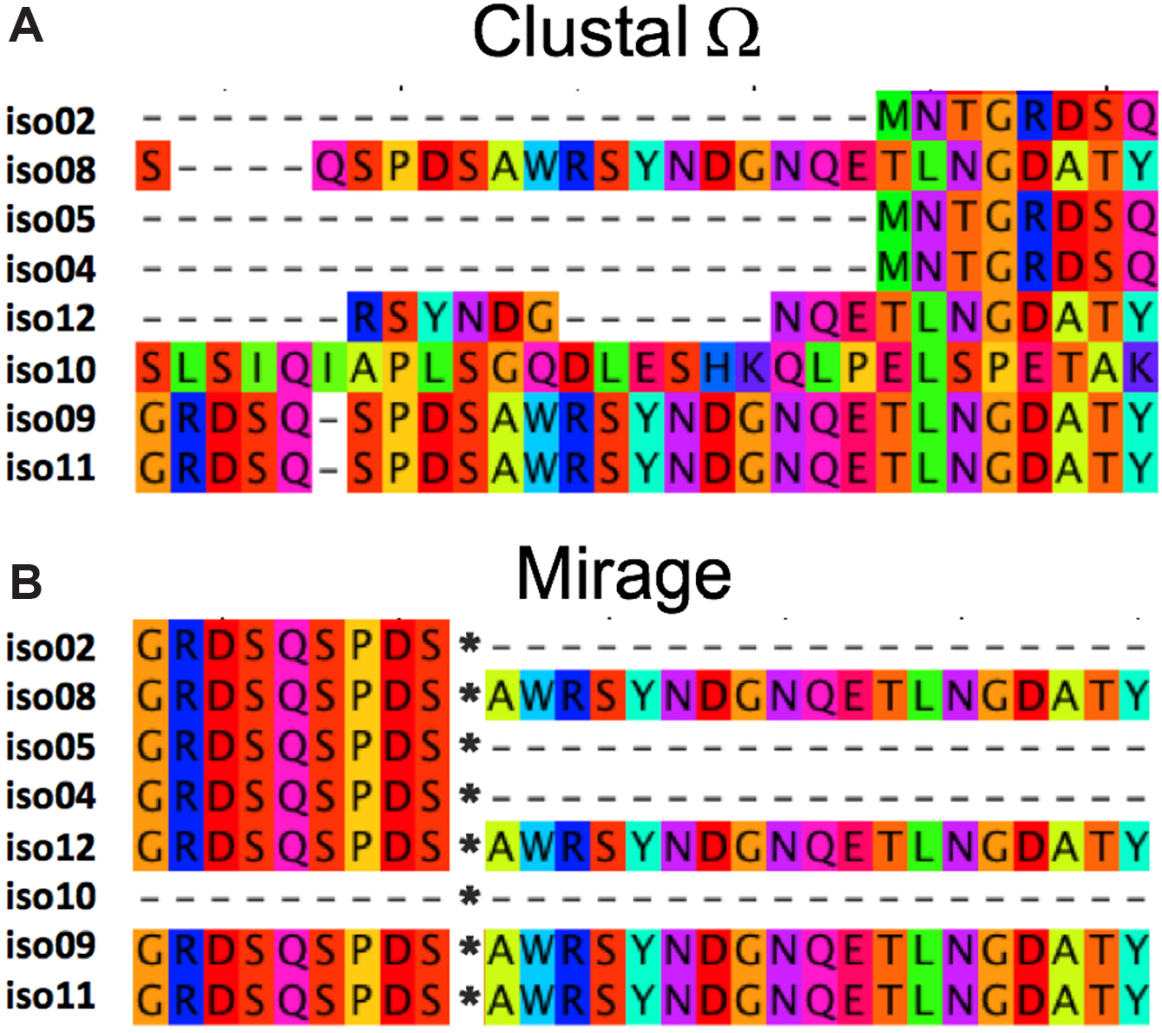
Multiple sequence alignments by (**A**) Clustal Ω and (**B**) Mirage (https://github.com/TravisWheelerLab/Mirage) of eight isoforms of SORBS2/ArgBP2 (O94875).

Protein families will be aligned separately using Mirage (29), a splice-aware MSA program that produces extremely accurate intra-species alignments. For example, Figure 6 compares alignments of eight isoforms of human ArgBP2 made with Clustal Ω (Figure 6A, 30) and Mirage (Figure 6B). Three isoforms in the Clustal alignment (isoforms 8, 9 and 11) are almost perfectly aligned; however, the remaining isoforms are mismatched at all residues. In contrast, there are no mismatches in the alignment produced by Mirage. It is anticipated that this change will significantly improve the accuracy and speed of the curation and annotation process.

### Long term

#### Quantitative data viewer

We will build tools that will enable users to view quantitative MS2 data sets that have been curated into PSP. For example, we have explored visualizing large-scale quantitative data with Clustergrammer, a prototype interactive and web-accessible heat-map viewer (31), an example of which can be viewed here: https://maayanlab.github.io/cst_drug_treatment/lung_CL_phos_ratios_Y

#### Visualizing regulatory interactions

We will integrate a web visualization tool enabling users to visualize the PTM-centric regulatory data curated into PSP, including upstream and downstream network connections and molecular interactions regulated by changes in the state of specific post-translationally modified sites.

#### Prediction of upstream kinases and binding partners

We will integrate a kinase predictor into PSP that combines position-specific scoring matrices derived from position-specific scoring matrices (32) with the specificity profiles derived from the experimentally observed substrate motifs extracted from PSP.

## DISCUSSION

While we feel that the addition of disease associated missense mutations opens up a fertile area for investigations into the possible rewiring of signaling pathways by single amino acid mutations, there are other explanations why a PTMVar may disrupt signaling networks. One of them is that the variant has altered protein stability, leading to its proteasomal degradation, or inhibiting its molecular interactions required for proper network connectivity (33,34). It would be valuable to include a score into PSP representing the effects of each missense mutation on protein stability in the future.

The inclusion of disease variants with post-translational modifications, and their visualization with interactive lollipop plots, provides the tools for exploring the intersection of mutations, PTMs, diseases and pathways. We hope that the integration of these multiple dimensions will promote novel hypotheses, advancing our understanding of pathogenesis in human disease.

## Supplementary Material

Supplementary Data
